# Design and Selection of Machine Learning Methods Using Radiomics and Dosiomics for Normal Tissue Complication Probability Modeling of Xerostomia

**DOI:** 10.3389/fonc.2018.00035

**Published:** 2018-03-05

**Authors:** Hubert S. Gabryś, Florian Buettner, Florian Sterzing, Henrik Hauswald, Mark Bangert

**Affiliations:** ^1^Department of Medical Physics in Radiation Oncology, German Cancer Research Center (DKFZ), Heidelberg, Germany; ^2^Medical Faculty of Heidelberg, Heidelberg University, Heidelberg, Germany; ^3^Heidelberg Institute for Radiation Oncology (HIRO), Heidelberg, Germany; ^4^Institute of Computational Biology, Helmholtz Zentrum München, Neuherberg, Germany; ^5^Clinical Cooperation Unit Radiation Oncology, German Cancer Research Center (DKFZ), Heidelberg, Germany; ^6^Department of Radiation Oncology, Heidelberg University Hospital, Heidelberg, Germany

**Keywords:** radiotherapy, IMRT, NTCP, xerostomia, head and neck, machine learning, radiomics, dosiomics

## Abstract

**Purpose:**

The purpose of this study is to investigate whether machine learning with dosiomic, radiomic, and demographic features allows for xerostomia risk assessment more precise than normal tissue complication probability (NTCP) models based on the mean radiation dose to parotid glands.

**Material and methods:**

A cohort of 153 head-and-neck cancer patients was used to model xerostomia at 0–6 months (early), 6–15 months (late), 15–24 months (long-term), and at any time (a longitudinal model) after radiotherapy. Predictive power of the features was evaluated by the area under the receiver operating characteristic curve (AUC) of univariate logistic regression models. The multivariate NTCP models were tuned and tested with single and nested cross-validation, respectively. We compared predictive performance of seven classification algorithms, six feature selection methods, and ten data cleaning/class balancing techniques using the Friedman test and the Nemenyi *post hoc* analysis.

**Results:**

NTCP models based on the parotid mean dose failed to predict xerostomia (AUCs < 0.60). The most informative predictors were found for late and long-term xerostomia. Late xerostomia correlated with the contralateral dose gradient in the anterior–posterior (AUC = 0.72) and the right–left (AUC = 0.68) direction, whereas long-term xerostomia was associated with parotid volumes (AUCs > 0.85), dose gradients in the right–left (AUCs > 0.78), and the anterior–posterior (AUCs > 0.72) direction. Multivariate models of long-term xerostomia were typically based on the parotid volume, the parotid eccentricity, and the dose–volume histogram (DVH) spread with the generalization AUCs ranging from 0.74 to 0.88. On average, support vector machines and extra-trees were the top performing classifiers, whereas the algorithms based on logistic regression were the best choice for feature selection. We found no advantage in using data cleaning or class balancing methods.

**Conclusion:**

We demonstrated that incorporation of organ- and dose-shape descriptors is beneficial for xerostomia prediction in highly conformal radiotherapy treatments. Due to strong reliance on patient-specific, dose-independent factors, our results underscore the need for development of personalized data-driven risk profiles for NTCP models of xerostomia. The facilitated machine learning pipeline is described in detail and can serve as a valuable reference for future work in radiomic and dosiomic NTCP modeling.

## 1 Introduction

Radiotherapy is the main treatment for head-and-neck tumors. Incidental irradiation of salivary glands often impairs their function, causing dryness in the mouth (xerostomia). Xerostomia significantly reduces patients’ quality of life, leading to dental health deterioration, oral infections, and difficulties in speaking, chewing, and swallowing.

The Quantitative Analyses of Normal Tissue Effects in the Clinic (QUANTEC) group recommended sparing at least one parotid gland to a mean dose <20 Gy or both parotid glands to a mean dose <25 Gy (1). Large-cohort studies confirmed that the mean dose is a good predictor of xerostomia (2, 3). However, it has also been observed that the mean dose failed to recognize patients at risk in cohorts where the majority of patients had met the QUANTEC guidelines, although the prevalence of xerostomia was reduced (4–6).

In recent years, a number of studies have investigated various patient- and therapy-related factors in hope of more precise xerostomia predictions. These included the mean dose to submandibular glands and the oral cavity (5, 7–9), sparing of the parotid stem cells region (10), three-dimensional dose moments (4), CT image features (11, 12), patients’ T stage, age, financial status, education, smoking, etc. (4, 5, 8).

Moreover, there has been growing interest in the adoption of machine learning classifiers in NTCP modeling (13–15). Buettner et al. used Bayesian logistic regression together with dose-shape features to predict xerostomia in head-and-neck cancer patients (4). Support vector machines were employed to model radiation-induced pneumonitis (16). Ospina et al. predicted rectal toxicity following prostate cancer radiotherapy using random forests (17).

Nevertheless, despite the growing interest in data-driven methods, there have been no published studies so far systematically evaluating how different machine learning techniques can be used to address the challenges specific to NTCP modeling. These include class imbalance due to low prevalence rates, heterogeneous and noisy data, large feature spaces, irregular follow-up times, etc. A comparable work has already been presented in the fields of bioinformatics (18, 19) and radiomics (20). Such analysis is missing for NTCP modeling, although it seems especially relevant.

In this context, we examined associations between xerostomia and various features describing parotid shape (radiomics), dose shape (dosiomics), and demographic characteristics. Besides investigating the individual predictive power of the features, we comprehensively evaluated the suitability of seven machine learning classifiers, six feature selection methods, and ten data cleaning/class balancing algorithms for multivariate NTCP modeling. The obtained results were compared to mean-dose models and the morphological model proposed by Buettner et al. (4). Furthermore, we proposed a longitudinal approach for NTCP modeling that includes the time after treatment as a model covariate. Doing so, rather than binning the data around a certain time point, better reflects the underlying data due to often irregular follow-up times.

## 2 Materials and Methods

### 2.1 Patients

The retrospective patient cohort collected for this study comprised head-and-neck cancer patients treated with radiotherapy at Heidelberg University Hospital in years 2010–2015. After excluding patients with nonzero baseline xerostomia, replanning during the treatment, tumor in the parotid gland, second irradiation, second chemotherapy, or ion beam boost, the cohort consisted of 153 patients. Patient and tumor characteristics are listed in Table 1. The study was approved by the Ethics Committee of Heidelberg University.

**Table 1:** Patients and tumor characteristics.

	All	0–6 months			6–15 months			15–24 months		
		Grade 0	Grade 1	Grade 2	Grade 0	Grade 1	Grade 2	Grade 0	Grade 1	Grade 2
Total patients	153	17	87	30	19	99	13	15	53	9
Age										
Median	61	60	60	62	60	61	61	61	61	61
Q1–Q3	55–66	54–66	54–64	53–69	57–63	53–66	54–68	55–68	52–66	54–68
Range	29–82	44–78	29–82	43–80	49–75	29–82	43–74	47–80	39–78	41–80
Sex										
Female	37	5	19	7	6	24	2	2	9	4
Male	116	12	68	23	13	75	11	13	44	5
Tumor site										
Hypopharynx/larynx	37	7	20	7	7	20	2	3	15	0
Nasopharynx	12	0	8	2	2	8	1	0	5	0
Oropharynx	99	9	57	20	10	69	9	11	32	9
Other	5	1	2	1	0	2	1	1	1	0
Radiation modality										
IMRT	37	2	25	5	1	29	2	2	18	1
Tomotherapy	116	15	62	25	18	70	11	13	35	8
Ipsi parotid dose (Gy)										
Median	24.3	22.9	25.0	23.0	19.5	24.8	25.9	22.9	23.8	24.5
Q1–Q3	20.6–27.6	18.5–24.6	21.4–29.0	21.4–25.4	16.8–24.3	21.8–28.7	21.8–27.2	18.5–31.5	20.8–26.4	21.6–26.2
Range	0.4–63.4	0.4–36.0	7.4–61.4	4.6–59.0	0.4–32.9	4.6–61.4	17.3–63.4	0.4–51.4	4.6–46.0	17.3–63.4
Contra parotid dose (Gy)										
Median	19.9	19.4	20.3	19.6	15.6	20.5	20.4	12.7	19.7	20.1
Q1–Q3	15.4–23.1	13.1–21.8	15.2–23.8	16.5–22.0	10.3–20.7	16.3–23.8	19.8–23.1	5.2–17.9	16.3–23.7	16.4–22.3
Range	0.3–30.9	0.3–24.9	4.1–28.6	4.2–26.2	0.3–27.9	4.1–30.9	15.1–26.2	0.3–27.9	4.1–27.2	15.1–26.0

*The total number of patients differs among the groups due to the follow-up availability*.

### 2.2 End Points

For this study, we analyzed 693 xerostomia toxicity follow-up reports. We aimed to model moderate-to-severe xerostomia defined as grade 2 or higher according to Common Terminology Criteria for Adverse Effects (CTCAE) v4.03 (21). In 74% of cases, either CTCAE v3.0 or v4.03 grading scale was used. Dry mouth (xerostomia) definitions were the same in both versions so no inconsistency in grading was introduced. In case no score was provided but descriptive toxicity information was available, appropriate scores were assigned together with Heidelberg University Hospital clinicians. To minimize intra- and interobserver variability in this process, a set of rules in the form of a dictionary was introduced.

**Figure 1:**
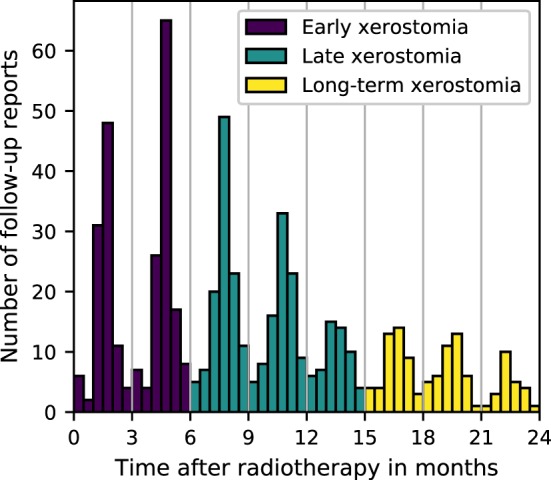
Frequency of the follow-up reports collection.

The follow-up reports were collected, on average, at 3-month intervals (Figure 1). The number of toxicity evaluations and the length of the follow-up varied from patient to patient. Due to the time-characteristic and the irregularity of the follow-up, two approaches were taken to model xerostomia: a time-specific approach and a longitudinal approach. In the time-specific approach, three time intervals were defined: 0–6, 6–15, and 15–24 months, to investigate early, late, and long-term xerostomia, respectively. In case there were multiple follow-up reports available for individual patients, the final toxicity score was calculated as the arithmetic mean rounded to the nearest integer number with x.5 being rounded up. In the longitudinal approach, no time-intervals were defined and no toxicity grades were averaged. Instead, each patient evaluation served as a separate observation and the time after treatment was included as a covariate in the model.

### 2.3 Features

The candidate xerostomia predictors comprised demographic, radiomic, and dosiomic features (Table 2). The radiomic and the dosiomic features were extracted from the CT- and the dose-cubes read from treatment planning DICOM files. In a preprocessing step, all the cubes were linearly interpolated to an isotropic 1 mm resolution. Moreover, we wanted to analyze the features in terms of ipsi- and contralateral rather than left and right parotid glands. This would, however, mean that certain spatial features would have either positive or negative value, depending on the tumor location (left or right). In order to solve that issue, the cubes were flipped through the sagittal plane for cases with the mean dose to the right parotid gland higher than the mean dose to the left parotid gland. All feature definitions were based on the LPS coordinate system, that is (right to left, anterior to posterior, inferior to superior). The detailed definitions of the features are provided in Appendix A.

**Table 2:** Feature sets before and after the removal of highly correlated pairs (Kendall’s |τ| > 0.5).

Feature group	Initial feature set	Final feature set
Demographics	Age, sex	Age, sex
Parotid shape	Volume, area, sphericity, eccentricity, compactness, λ*_1_*, λ*_2_*, λ*_3_*	Volume, sphericity, eccentricity
Dose–volume histogram	Mean, spread, skewness, D2, D98, D10, D20, D30, D40, D50, D60, D70, D80, D90, V10, V15, V20, V25, V30, V35, V40, V45, entropy, uniformity	Mean, spread, skewness
Subvolume mean dose	$s_{x}^{\text{1}}$, $s_{x}^{\text{2}}$, $s_{x}^{\text{3}}$, $s_{y}^{\text{1}}$, $s_{y}^{\text{2}}$, $s_{y}^{\text{3}}$, $s_{z}^{\text{1}}$, $s_{z}^{\text{2}}$, $s_{z}^{\text{3}}$	
Spatial dose gradient	Gradient_x_, gradient_y_, gradient_z_	Gradient_x_, gradient_y_, gradient_z_
Spatial dose spread	*η*_200_, *η*_020_, η_002_	*η*_200_, *η*_020_, *η*_002_
Spatial dose correlation	*η*_110_, *η*_101_, *η*_011_	*η*_110_, *η*_101_, *η*_011_
Spatial dose skewness	*η*_300_, *η*_030_, *η*_003_	*η*_300_, *η*_030_, *η*_003_
Spatial dose coskewness	*η*_012_, *η*_021_, *η*_120_, *η*_102_, *η*_210_, *η*_201_	*η*_012_, *η*_021_, *η*_120_, *η*_102_, *η*_210_, *η*_201_

*Feature definitions are provided in Appendix A*.

To reduce feature redundancy, the Kendall rank correlation coefficient was calculated for all feature pairs. Kendall’s τ allows to measure ordinal association between two features, that is agreement in ranks assigned to the observations. It can be interpreted as a difference between the probability that both features rank a random pair of observations in the same way and the probability that they rank these observations in a different way (22). We considered feature pairs with |τ| > 0.5 in both glands as highly correlated and suitable for rejection from the feature set. This arbitrarily chosen threshold corresponds to a 75% probability that the two features rank a random pair of observations in the same way. Whenever a pair of features was found highly correlated, we decided to keep the feature that was conceptually and computationally simpler, e.g., mean dose over Dx, parotid volume over parotid compactness, etc.

### 2.4 Previously Proposed NTCP Models

Logit and probit NTCP models based on the mean dose to parotid glands have been extensively used in modeling xerostomia (2, 3, 23, 24). We have tested four different mean-dose models to evaluate predictive power of the mean dose in our cohort: three univariate logistic regression models based on the ipsilateral mean dose, the contralateral mean dose, and the mean dose to both parotid glands, as well as one bivariate logistic regression model based on the mean dose to contralateral and to ipsilateral parotid glands.

As an alternative to the mean-dose models, Buettner et al. (4) proposed a multivariate logistic regression model based on three-dimensional dose moments to predict xerostomia. The model was retrained and tested on our data set.

### 2.5 Univariate Analysis

The univariate analysis was performed to investigate associations of single features with the outcome at different time intervals. First, all features were normalized *via* Z-score normalization to zero mean and unit variance. Next, for each feature, the Mann–Whitney *U* statistic was calculated. The area under the receiver operating characteristic curve (AUC) is directly related to the *U* statistic and follows from the formula $\mathrm{AUC}=\frac{U}{n_{-}n_{+}}$, where *n_−_*and *n_+_* are the size of the negative and the size of the positive class, respectively (25). For all AUCs, 95% confidence intervals were estimated by bias-corrected and accelerated (BCa) bootstrap (26). The number of type I errors, that is falsely rejected null hypotheses, was controlled with the false discovery rate (FDR). The FDR is defined as the expected proportion of true null hypotheses in the set of all the rejected hypotheses (27). We applied the Gavrilov-Benjamini-Sarkar procedure to bound the FDR ≤ 0.05 (28). Additionally, for each feature, univariate logistic regression models were fitted and tolerance values corresponding to 20% (TV20), 10% (TV10), and 5% (TV5) complication probability were calculated.

### 2.6 Multivariate Analysis

**Figure 2:**
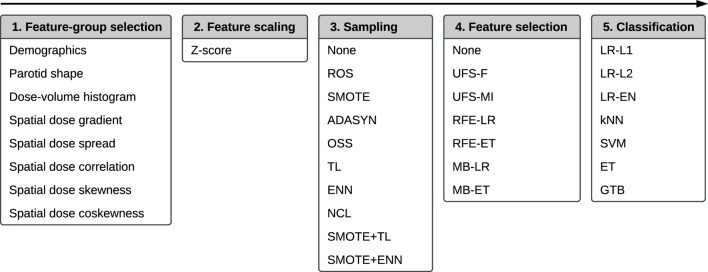
The workflow of a multivariate five-step model building comprising, in this order, feature-group selection, feature scaling, sampling, feature selection, and classification.

The multivariate analysis allowed to examine interactions between the features and their relative relevancy and redundancy. It was a multi-step process comprising feature-group selection, feature scaling, sampling (data cleaning and/or class balancing), feature selection, and classification. The workflow is presented in Figure 2.

#### 2.6.1 Workflow

The first step of the workflow was a random selection of the feature-groups (Table 2) used for model training. It allowed for an initial, unsupervised dimensionality reduction of the feature space, which typically translates into an improved predictive performance and a more straightforward interpretation of the models. The selection was realized by performing a Bernoulli trial for every feature group with a 50% chance of success. If a given group was selected, all features belonging to this group were accepted for further analysis. If no group was selected after performing all Bernoulli trials, the procedure was repeated for all feature groups.

In the second step, all features were scaled *via* Z-score normalization. Normalization of the features often improves stability and speed of optimization algorithms.

The third step served the purpose of class balancing and data cleaning. A class imbalance, noise, and a small size of the minority class can negatively affect the performance of a predictive model (29, 30). We investigated whether sampling methods designed to reduce noise and improve definitions of class clusters could enhance model performance. Ten algorithms were examined: random oversampling (ROS), synthetic minority oversampling (SMOTE), adaptive synthetic sampling (ADASYN), one-sided selection (OSS), Tomek links (TL), the Wilson’s edited nearest neighbor rule (ENN), the neighborhood cleaning rule (NCL), synthetic minority oversampling followed by the Wilson’s edited nearest neighbor rule (SMOTE + ENN), and synthetic minority oversampling followed by Tomek links (SMOTE + TL). The detailed description of the sampling algorithms is given in Appendix B.

The fourth step of the analysis was feature selection. The rationale for feature selection is a reduction of model complexity, which facilitates understanding of the relations between the predictors and the modeled outcome (here: xerostomia) (31). In this study, we tested six feature selection algorithms: univariate feature selection by F-score (UFS-F), univariate feature selection by mutual information (UFS-MI), recursive feature elimination by logistic regression (RFE-LR), recursive feature elimination by extra-trees (RFE-ET), model-based feature selection by logistic regression (MB-LR), and model-based feature selection by extra-trees (MB-ET). The details on the feature selection algorithms are provided in Appendix C.

The last step of the workflow was classification. We compared seven classification algorithms: logistic regression with L1 penalty (LR-L1), logistic regression with L2 penalty (LR-L2), logistic regression with elastic net penalty (LR-EN), k-nearest neighbors (kNN), support vector machines (SVM), extra-trees (ET), and gradient tree boosting (GTB). A more detailed description of the classification algorithms is given in Appendix D.

The models were build for every combination of the classification, feature selection, and sampling algorithms. This resulted in 490 models per end point or 1,960 models in total. A given classifier or a feature selection algorithm was involved in 210 time-specific and 70 longitudinal models. Every sampling method was part of 147 time-specific and 49 longitudinal models.

#### 2.6.2 Model Tuning

In the process of model building every model was tuned, that is its hyperparameters were optimized to maximize the prediction performance. The type and the range of the hyperparameters were based on previously reported values that worked well in various machine learning tasks (Appendices B, C, and D).

For each model, the hyperparameter optimization was realized by a random search (32). First, 300 random samples were selected from the hyperparameter space. Secondly, for each hyperparameter sample, the model performance was evaluated using cross-validation. Lastly, the model was retrained using all data with the hyperparameter configuration that maximized the cross-validated AUC.

In the time-specific models, the cross-validation was done by the stratified Monte Carlo cross-validation (MCCV) (33) with 300 splits and 10% of observations held out for testing at each split. For the longitudinal models, we used modified leave-pair-out cross-validation (LPOCV) (34, 35). In our LPOCV implementation, all the training observations sharing patient ID with the test fold observations were removed at each split. This decision was motivated by the fact that the observations sharing patient ID differ only in the time of the follow-up evaluation; not removing them from the training fold would lead to overoptimistic performance scores. Additionally, instead of all possible positive–negative pairs, as in typical LPOCV, only a random subset of 300 positive–negative pairs was used. This allowed for a reduction of the computation time. Confidence intervals for the model tuning AUC estimates were calculated with BCa bootstrap.

#### 2.6.3 Comparison of Machine Learning Algorithms

In order to compare the algorithms in terms of their influence on the average predictive performance of the model, we looked at the classifiers, the feature selection algorithms, and the sampling methods separately. Additionally, the analysis was performed independently for the time-specific and the longitudinal models.

The statistical significance of the differences between the algorithms was evaluated by the Friedman test followed by the Nemenyi *post hoc* analysis. The Friedman test computes average performance ranks of the algorithms and tests whether they have the same influence on the AUC score of the model. If the null hypothesis was rejected, we proceeded with the *post hoc* analysis. With the Nemenyi *post hoc* test, we calculated the critical difference at a significance level of 0.05. When the average performance ranks of two algorithms differed by at least the critical difference, they were significantly different.

As mentioned before, this analysis was repeated six times to test the classifiers, the feature selection algorithms, and the sampling methods separately in the time-specific and the longitudinal models. Therefore, the Holm–Bonferroni method was used to control the family-wise error rate (FWER) of the Friedman tests, that is the probability of making at least one incorrect rejection of a true null hypothesis in any of the comparisons (36). The significance level for the FWER was set to 0.05.

#### 2.6.4 Generalization Performance

Hyperparameter optimization comes at a cost. On the one hand, it allows to tune the model so it fits well the underlying data. On the other hand, the performance of the tuned model may be overoptimistic due to a favorable selection of hyperparameters. In order to estimate the generalization performance of a model, that is its performance on new, unseen data, the data used for model tuning must be separate from the data used for model testing. Due to the modest size of our data set, instead of dividing the data to training, validation, and test folds, we decided to test the models using nested-cross validation (37).

Nested cross-validation is essentially cross validation within cross validation. Part of the data is set aside for testing and the rest is used for model tuning (as described in the previous section). Next, the tuned model is tested on the part of data previously set aside for testing. Then, the procedure is repeated, that is another randomly selected part of the data is set aside for testing and the rest is used for model tuning. This is repeated until the desired number of iterations is achieved.

Unfortunately, due to high computation cost, it was not feasible to calculate the expected generalization performance of all 1,960 models. Therefore, the models were first stratified by end point and classifier, and then nested cross-validation was conducted for the best performing models. The inner loops of the nested cross-validation, which were responsible for model tuning, were the same as described in Section 2.6.2. The outer loops were realized by the MCCV with 100 splits and a 10% test fold (time-specific models) or the modified LPOCV (longitudinal models). Confidence intervals for the generalization AUCs were calculated with BCa bootstrap.

### 2.7 Software

The MATLAB code used for DICOM import, processing, and feature extraction was made publicly available on GitHub (https://github.com/hubertgabrys/DicomToolboxMatlab). For visualization, statistical analysis, model building, and model testing, the following open-source Python packages were used: imbalanced-learn (38), Matplotlib (39), NumPy & SciPy (40), Orange (41), Pandas (42), scikit-learn (43), scikits-bootstrap, and XGBoost (44).

## 3 Results

### 3.1 Feature Correlations

After removing the features correlated with the mean dose, the skewness of the dose–volume histogram, and the parotid volume, there were no highly correlated feature pairs left. The remaining features are listed in Table 2.

### 3.2 Mean-Dose and Morphological Models

The predictive performance scores of the mean-dose models and the morphological model are presented in Table 3. The mean-dose models failed to predict xerostomia (AUC < 0.60) at all time-intervals as well as in the longitudinal approach. The morphological model achieved fair performance (AUC = 0.64) only in predicting long-term xerostomia.

**Table 3:** Predictive performance of the mean-dose models and the morphological model proposed by Buettner et al. (4), that is logistic regression with $\eta_{111}^{\text{i}}$, $\eta_{002}^{\text{c}}$, $\eta_{300}^{\text{c}}$, and $\eta_{110}^{\text{i}}$$\eta_{110}^{\text{c}}$.

End point	Model	AUC
Early	Mean^i^	0.58 (0.56–0.60)
	Mean^c^	0.42 (0.41–0.44)
	Mean^b^	0.50 (0.48–0.53)
	Mean^i^, mean^c^	0.49 (0.48–0.51)
	Morphological	0.42 (0.40–0.44)

Late	Mean^i^	0.48 (0.44–0.51)
	Mean^c^	0.58 (0.55–0.61)
	Mean^b^	0.55 (0.52–0.58)
	Mean^i^, mean^c^	0.54 (0.51–0.57)
	Morphological	0.59 (0.56–0.62)

Long-term	Mean^i^	0.40 (0.37–0.44)
	Mean^c^	0.58 (0.55–0.61)
	Mean^b^	0.56 (0.52–0.60)
	Mean^i^, mean^c^	0.47 (0.44–0.50)
	Morphological	0.64 (0.60–0.67)

Longitudinal	Mean^i^	0.51 (0.45–0.56)
	Mean^c^	0.57 (0.51–0.62)
	Mean^b^	0.50 (0.44–0.55)
	Mean^i^, mean^c^	0.52 (0.46–0.58)
	Morphological	0.55 (0.49–0.60)

*i, ipsilateral gland; c, contralateral gland; b, both glands*.

### 3.3 Univariate Analysis

**Figure 3:**
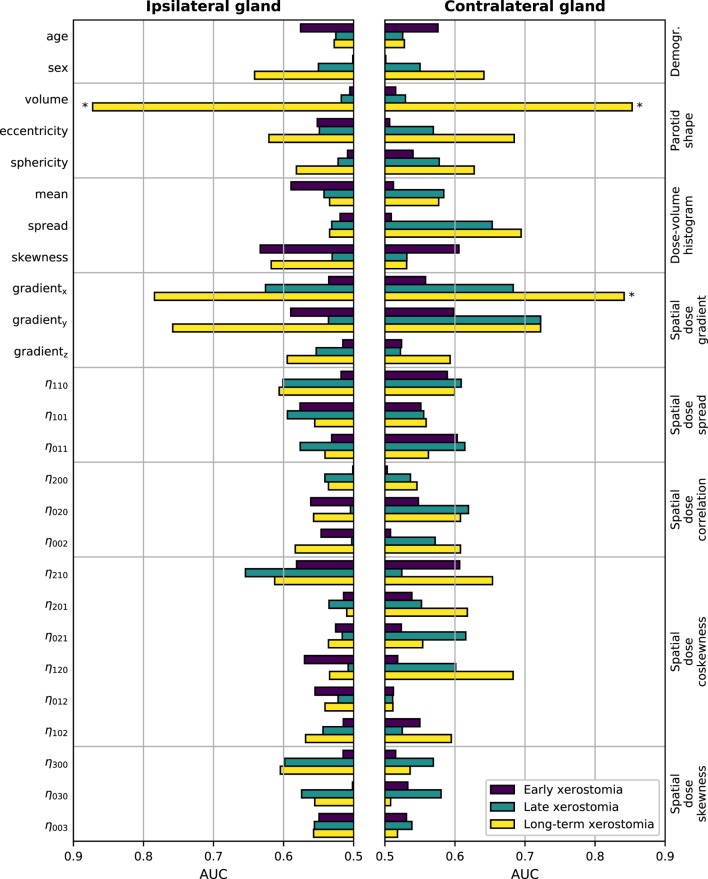
Predictive power of individual features in the time-specific models measured with the area under the receiver operating characteristic curve (AUC). The left-hand side vertical axis lists the features, the right-hand side vertical axis lists the feature groups. The AUCs were calculated from the corresponding Mann–Whitney *U* statistic. Bars marked with * are significant at the false discovery rate (FDR) ≤ 0.05.

The results of the univariate analysis are presented in Figure 3. There was little association between single predictors and xerostomia within the first six months after treatment. Late xerostomia correlated with individual features slightly better. The most informative were contralateral dose gradients in the right–left direction (AUC = 0.68 (0.53–0.82)) and the anterior–posterior direction (AUC = 0.72 (0.58–0.84)). Nevertheless, the AUCs were too low to be statistically significant at the FDR ≤ 0.05. Long-term xerostomia was predicted well by parotid volumes, right–left dose gradients, and anterior–posterior dose gradients. Three models were statistically significant at the FDR ≤ 0.05: the ipsilateral parotid volume (AUC = 0.87 (0.75–0.95), TV20 = 9,894 mm^3^, TV10 = 15,681 mm^3^, TV5 = 21,014 mm^3^), the contralateral parotid volume (AUC = 0.85 (0.66–0.98), TV20 = 9,169 mm^3^, TV10 = 14,533 mm^3^, TV5 = 19,475 mm^3^), and the contralateral gradient in the right–left direction (AUC = 0.84 (0.71–0.93), TV20 = 1.49 Gy/mm, TV10 = 1.29 Gy/mm, TV5 = 1.10 Gy/mm). Statistical significance of three tests at the FDR ≤ 0.05 translates into a 85.7% and a 99.3% lower bound on the probability that all three tests are truly positive or that at most one test is falsely positive, respectively.

**Figure 4:**
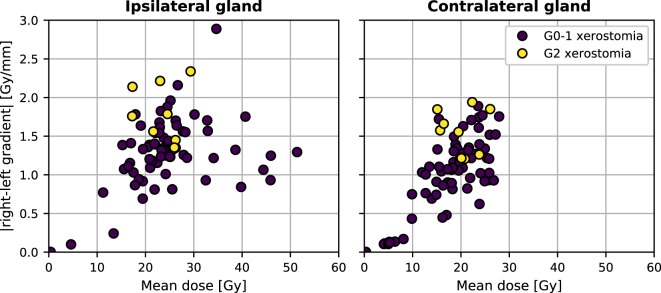
The mean dose and the absolute right–left dose gradient distribution in our patient cohort.

Neither the mean dose to the contralateral nor the mean dose to the ipsilateral parotid gland discriminated well between patients with and without xerostomia in the time-specific and the longitudinal approach. Figure 4 shows the comparison between the mean dose and the absolute right–left dose gradient values for the patients with long-term xerostomia.

### 3.4 Comparison of Classification, Feature Selection, and Sampling Algorithms

**Figure 5:**
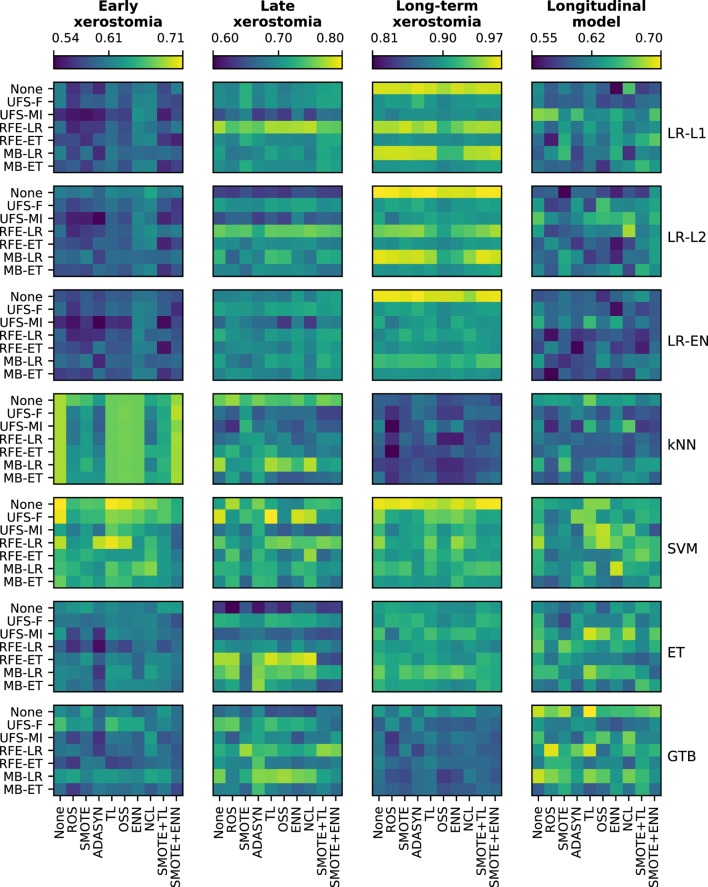
A comparison of classification, feature selection, and sampling algorithms in terms of their predictive performance in model tuning. All heat maps in a given column belong to a single end point, whereas all heat maps in a given row correspond to a single classifier. In each heat map, rows represent feature selection algorithms and columns correspond to sampling methods. The color maps are normalized per end point. The color bar ticks correspond to the worst, average, and the best model performance.

There was a clear difference in the average performance between early (AUC ≈ 0.60), late (AUC ≈ 0.70), and long-term (AUC ≈ 0.90) xerostomia models (Figure 5). After applying the Holm-Bonferroni correction, all the Friedman tests were significant at the FWER ≤ 0.05. Therefore, classification, feature selection, and sampling algorithms were compared for both the time-specific and the longitudinal models.

**Figure 6:**
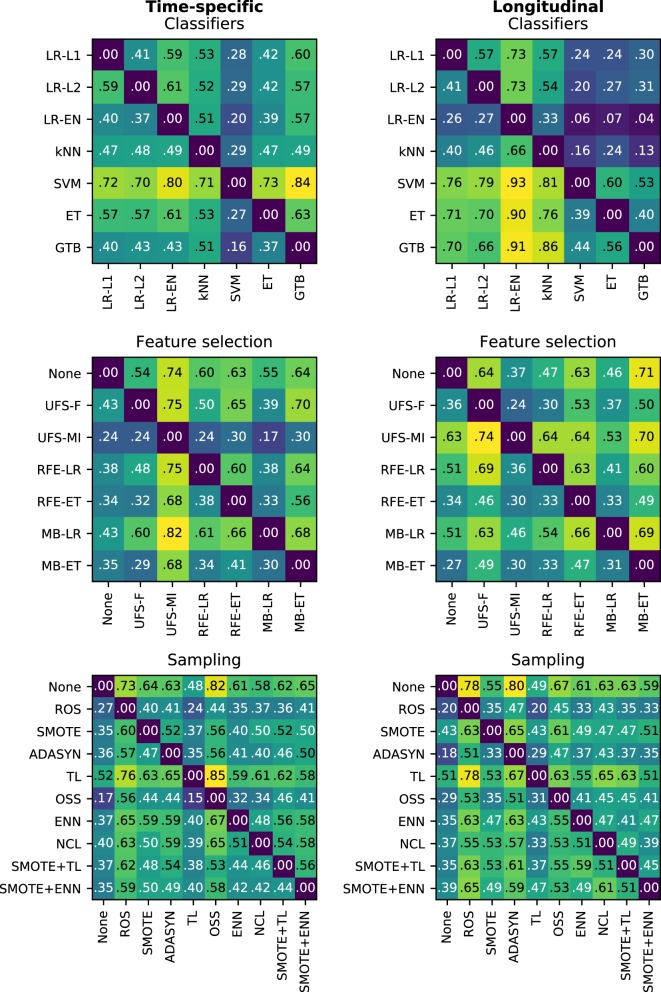
Heat maps showing a proportion of times a given algorithm on the vertical axis outperformed another algorithm on the horizontal axis in terms of the best AUC in model tuning. For example, support vector machines (SVM) performed better than extra-trees (ET) in 73% of the time-specific models.

**Figure 7:**
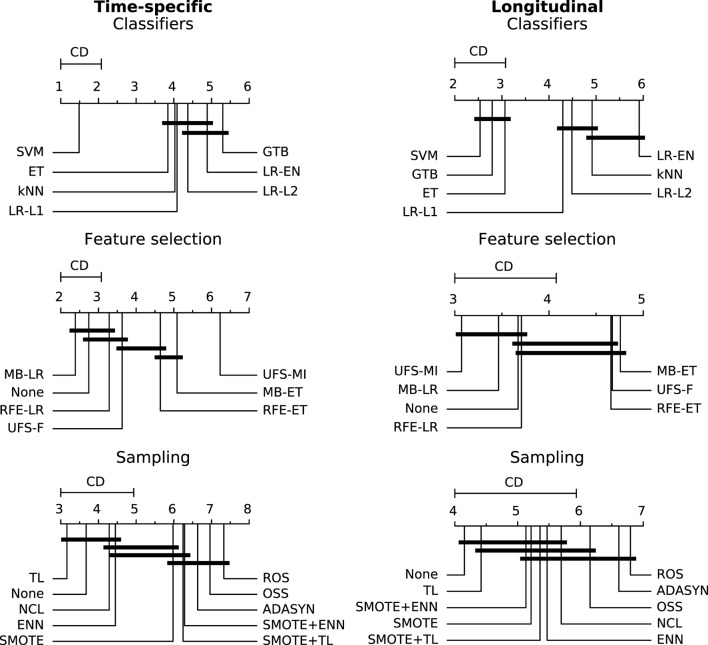
A comparison of classification, feature selection, and sampling methods against one another with the Nemenyi test. Lower ranks correspond to better performance of the algorithm, that is rank 1 is the best. Algorithms which ranks differ by less than the critical difference (CD) are not significantly different at 0.05 significance level and are connected by the black bars.

In the time-specific models, the support vector machine was by far the best scoring classifier, outperforming the other classifiers in over 70% of cases (Figure 6), whereas gradient tree boosting was on average the worst performing classifier (Figure 7). Conversely, gradient tree boosting together with support vector machines and extra-trees predicted xerostomia significantly better than all the other classifiers in the longitudinal approach.

The logistic regression-based algorithms performed significantly better than the feature selection methods based on extra-trees, in both the time-specific and the longitudinal models. Interestingly, while univariate feature selection by mutual information was the worst performing feature selection method in the time-specific models, it was one of the best in the longitudinal approach. Not performing feature selection was not disadvantageous in terms of predictive performance.

In both the time-specific and the longitudinal approach, no sampling algorithm gave a significant advantage over no sampling at all. In the time-specific models, Tomek links and the neighborhood cleaning rule performed significantly better than any oversampling algorithm. In the longitudinal models, Tomek links performed significantly better than random oversampling or ADASYN.

### 3.5 Generalization Performance

The best performing models stratified by end point and classifier are listed in Table 4. These models were retested by nested cross-validation to estimate their generalization performance. Early xerostomia (0–6 months after treatment) was predicted fairly well only by the k-nearest neighbors classifier (AUC = 0.65). The models of late xerostomia (6–15 months after treatment) generalized slightly better with logistic regression, k-nearest neighbors, and gradient tree boosting scoring AUC > 0.60. For long-term xerostomia (15–24 months after treatment), the models generalized best with the AUC ranging from 0.74 (k-nearest neighbors) to 0.88 (extra-trees). The longitudinal models failed to generalize except the gradient tree boosting classifier, which achieved AUC = 0.63. Generalization AUCs were on average 0.10 lower than tuning AUCs for all the analyzed end points.

**Table 4:** Expected generalization performance of selected models evaluated by nested cross-validation.

End point	Classifier	Feature selection	Sampling	AUC tuning	AUC testing
Early	LR-L1	RFE-ET	NCL	0.62 (0.60–0.64)	0.56 (0.53–0.60)
	LR-L2	RFE-LR	NCL	0.62 (0.60–0.64)	0.46 (0.42–0.49)
	LR-EN	MB-ET	NCL	0.62 (0.60–0.64)	0.54 (0.50–0.57)
	kNN	UFS-F	SMOTE + ENN	0.68 (0.66–0.70)	0.65 (0.62–0.68)^a^
	SVM	UFS-F	None	0.70 (0.68–0.72)	0.57 (0.53–0.61)
	ET	MB-LR	NCL	0.63 (0.61–0.65)	0.44 (0.41–0.47)
	GTB	UFS-F	None	0.66 (0.64–0.68)	0.55 (0.51–0.59)

Late	LR-L1	RFE-LR	NCL	0.78 (0.75–0.80)	0.63 (0.56–0.69)
	LR-L2	RFE-LR	NCL	0.76 (0.73–0.78)	0.60 (0.53–0.66)
	LR-EN	MB-LR	SMOTE + TL	0.73 (0.70–0.76)	0.56 (0.51–0.62)
	kNN	MB-LR	NCL	0.78 (0.76–0.80)	0.62 (0.57–0.67)
	SVM	UFS-F	TL	0.80 (0.77–0.82)	0.52 (0.46–0.58)
	ET	RFE-ET	NCL	0.78 (0.75–0.80)	0.55 (0.50–0.61)
	GTB	MB-LR	OSS	0.77 (0.75–0.79)	0.65 (0.59–0.70)^a^

Long-term	LR-L1	MB-LR	ROS	0.95 (0.94–0.96)	0.86 (0.80–0.90)
	LR-L2	MB-LR	None	0.96 (0.95–0.97)	0.86 (0.81–0.90)
	LR-EN	MB-LR	SMOTE + ENN	0.92 (0.90–0.93)	0.83 (0.76–0.88)
	kNN	UFS-MI	TL	0.88 (0.86–0.90)	0.74 (0.68–0.80)
	SVM	RFE-LR	ENN	0.94 (0.92–0.96)	0.79 (0.73–0.85)
	ET	MB-LR	ENN	0.93 (0.92–0.94)	0.88 (0.84–0.91)^a^
	GTB	UFS-F	ROS	0.89 (0.86–0.91)	0.77 (0.71–0.83)

Longitudinal	LR-L1	UFS-MI	None	0.63 (0.57–0.68)	0.52 (0.41–0.61)
	LR-L2	RFE-LR	NCL	0.60 (0.55–0.66)	0.39 (0.29–0.48)
	LR-EN	UFS-MI	TL	0.62 (0.57–0.68)	0.52 (0.42–0.60)
	kNN	UFS-MI	NCL	0.65 (0.61–0.69)	0.58 (0.49–0.66)
	SVM	UFS-MI	OSS	0.66 (0.60–0.71)	0.57 (0.46–0.66)
	ET	UFS-MI	TL	0.66 (0.61–0.71)	0.51 (0.40–0.60)
	GTB	RFE-LR	ROS	0.68 (0.62–0.72)	0.63 (0.52–0.71)^a^

*^a^Best performing models at a given end point*.

### 3.6 Model Interpretation

**Figure 8:**
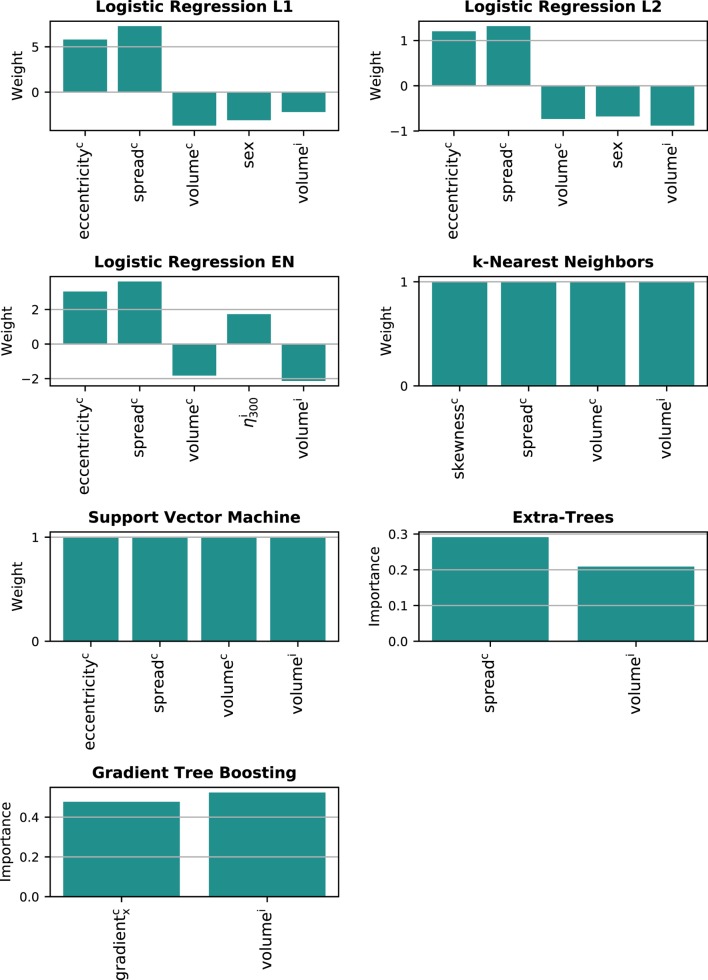
Features underlying the multivariate models of long-term xerostomia. i, ipsilateral gland; c, contralateral gland.

Only the models predicting long-term xerostomia achieved high generalization scores, that is AUC > 0.70. For that reason, model interpretation was performed only for this end point. The multivariate models of long-term xerostomia relied mostly on the parotid gland volume, the spread of the contralateral dose–volume histogram, and the parotid gland eccentricity (Figure 8). The contralateral dose gradient in the right–left direction, despite good univariate predictive power, was included in only one model.

## 4 Discussion

The univariate analysis showed that parotid- and dose-shape features can be highly predictive of xerostomia. Patients with small parotid glands (median parotid volume in the positive group 9,557 vs. 14,374 mm^3^ in the negative group) and steep dose gradients in the patient’s right–left direction (median gradient in the positive group 1.7 vs. 1.2 Gy/mm in the negative group) were significantly more likely to develop long-term xerostomia. A possible explanation of this finding could be the fact that parotid glands typically shrink and move toward the medial direction during the course of radiotherapy. As a result, for patients with small parotid glands, the gradient is a proxy for the change of any dose-related metric subject to motion. As such, this might be an indicator of neglected motion and deformation effects during the modeling process.

Nevertheless, good discriminative power of the dose gradients and poor performance of the mean dose should be put into perspective of the previous studies validating mean-dose models. In cohorts where patients received a high radiation dose to parotid glands, the mean dose allowed achieving AUC above 0.80 (2, 3). It seems that inclusion of patients with less conformal treatment plans and a higher dosage to parotids would result in a cluster of patients with complications in the high-dose region of Figure 4. Therefore, for relatively high doses, the mean dose alone is a good xerostomia predictor irrespective of the dose gradient, whereas in the low-dose regime of modern radiotherapy treatments dose gradients are more informative and the mean dose is less predictive.

In the multivariate analysis, we did not find a model that would achieve generalization AUC above 0.65 for early or late-effects, even though a few univariate models of late xerostomia exceeded that value. Similarly, the multivariate models of long-term xerostomia, despite their good generalization scores (AUC_max_ = 0.88), performed on a par with the univariate models based on the parotid volume or the contralateral dose gradient in the patient’s right–left direction. Comparable performance of the univariate and the multivariate models could be caused by the small sample size, especially the small minority class. In such setting, the distribution of model covariates can nonnegligibly differ between training and testing folds, hindering model training and reducing performance of the model.

The analysis of the multivariate models highlighted the importance of personalized treatment planning in radiotherapy. The models were strongly based on patient-specific and dose-independent features, such as parotid volume, parotid eccentricity, and the patient’s sex. Females with small, elongated parotid glands were at higher risk of long-term xerostomia than males with large and rather round parotids. Interestingly, the dose gradient, despite relatively high predictive power, was included in only one model. Instead, the most common dosiomic feature was the spread of the contralateral dose–volume histogram quantifying the SD of the dose within a parotid gland. Nevertheless, due to the geometry of the problem, the DVH spread and spatial dose gradients measured a similar characteristic of the dose distribution. That is, a large spread of the DVH was present when part of the parotid gland received high dose, whereas another part was spared.

In the time-specific models, the support vector machine was most commonly the best classifier. The other classifiers performed similarly to one another. The unexceptional performance of the ensemble methods (extra-trees and gradient tree boosting) could stem from the fact that complex models need more training samples to correctly learn the decision boundary. Among the longitudinal models, we saw a more commonly observed classifier “ranking,” that is GTB > ET > SVM > LR > kNN (19). Feature selection did not give a clear advantage over no feature selection in terms of the predictive performance. Nonetheless, feature selection allowed for a reduction of model complexity and made model interpretation easier. The best results were achieved with the logistic regression-based algorithms and feature selection by mutual information (only in the longitudinal models). We have not found evidence that sampling methods improve accuracy of predictions. Moreover, we observed that certain kinds of sampling, especially random oversampling, can significantly decrease predictive performance of the models.

Nested cross-validation proved to be an important step in the analysis. On average, the generalization AUCs were significantly lower than the AUCs achieved in model tuning. Our findings confirm the notion that single cross-validation can lead to overoptimistic performance estimates when hyperparameter tuning is involved in model building.

## 5 Conclusion

We demonstrated that in a highly conformal regime of modern radiotherapy, use of organ- and dose-shape features can be advantageous for modeling of treatment outcomes. Moreover, due to strong dependence on patient-specific factors, such as the parotid shape or the patient’s sex, our results highlight the need for development of personalized data-driven risk profiles in future NTCP models of xerostomia.

Our results show that the choice of a classifier and a feature selection algorithm can significantly influence predictive performance of the NTCP model. Moreover, in relatively small clinical data sets, simple logistic regression can perform as well as top-ranking machine learning algorithms, such as extra-trees or support vector machines. We saw no significant advantage in using data cleaning or reducing the class imbalance. Our study confirms the need for significantly larger patient cohorts to benefit from advanced classification methods, such as gradient tree boosting. We showed that single cross-validation can lead to overoptimistic performance estimates when hyperparameter optimization is involved; either nested cross-validation or an independent test set should be used to estimate the generalization performance of a model.

## List of Non-Standard Abbreviations

**Table:** 

**Classification**	
LR-L1	Logistic regression with L1 penalty
LR-L2	Logistic regression with L2 penalty
LR-EN	Logistic regression with elastic net penalty
kNN	k-Nearest neighbors
SVM	Support vector machine
ET	Extra-trees
GTB	Gradient tree boosting
**Feature selection**	
UFS-F	Univariate feature selection by F-score
UFS-MI	Univariate feature selection by mutual information
RFE-LR	Recursive feature elimination by logistic regression
RFE-ET	Recursive feature elimination by extra-trees
MB-LR	Model-based feature selection by logistic regression
MB-ET	Model-based feature selection by extra-trees
**Sampling**
ROS	Random oversampling
SMOTE	Synthetic minority
	oversampling
ADASYN	Adaptive synthetic sampling
OSS	One-sided selection
TL	Tomek links
ENN	Wilson’s edited nearest
	neighbor rule
NCL	Neighborhood cleaning rule
SMOTE + ENN	SMOTE followed by the ENN
SMOTE + TL	SMOTE followed by TL

## Ethics Statement

The study was conducted in accordance with the Declaration of Helsinki and was approved by the Ethics Committee of Heidelberg University. Nr. S-392/2016 “Validation and development of probabilistic prediction models for radiation-induced xerostomia.”

## Author Contributions

HG, FS, HH, and MB contributed to the acquisition of the clinical data. HG, FS, and MB contributed to the analysis of the follow-up data. HG, FB, and MB contributed to the methodology. HG performed feature extraction, data visualization, statistical analysis, and drafted the manuscript. MB was the senior author supervising the project.

## Conflict of Interest Statement

The authors declare that the research was conducted in the absence of any commercial or financial relationships that could be construed as a potential conflict of interest.

## Appendix A

The MATLAB code used for feature extraction is available on GitHub https://github.com/hubertgabrys/DicomToolboxMatlab.

### A Parotid Shape

#### A.1 Volume

Volume *V* of the parotid gland.

#### A.2 Surface area

Surface area *A* of the parotid gland.

#### A.3 Sphericity

Parotid gland sphericity was defined as the ratio of the surface area of a sphere of the same volume as the parotid gland to the actual surface area of the parotid

$$\Psi =\frac{\pi^{\frac{1}{3}}{\left(6V\right)}^{\frac{2}{3}}}{A}.$$

#### A.4 Compactness

Parotid gland compactness was defined as a ratio of the parotid gland surface area to the parotid gland volume.

$$\kappa =\frac{A}{V}.$$

#### A.5 Eccentricity

Eccentricity *ε* measured how elongated the parotid gland was. Larger asymmetry of the gland corresponded to larger values of *ε*.

$$\varepsilon =1-\sqrt{\frac{\lambda_{\min}}{\lambda_{\max}}},$$

where eigenvalues λ*_i_* of the parotid shape covariance matrix correspond to the dimensions of the parotid gland along the principal axes defined by the eigenvectors. The covariance matrix is defined as:

$$\text{Cov}\left[I\left(x,y,z\right)\right]=\left(\begin{array}{ccc} \mu_{200} & \mu_{110} & \mu_{101}\\ \mu_{110} & \mu_{020} & \mu_{011}\\ \mu_{101} & \mu_{011} & \mu_{002} \end{array}\right)$$

$$\mu_{\mathrm{pqr}}=\underset{x,y,z}{\sum }\;{\left(x-\bar{x}\right)}^{p}{\left(y-ȳ\right)}^{q}{\left(z-\bar{z}\right)}^{r}I\left(x,y,z\right),$$

$$\bar{x}=\frac{\sum_{x,y,z}\;\mathrm{xI}\left(x,y,z\right)}{\sum_{x,y,z}\;I\left(x,y,z\right)}$$

where *x*, *y*, and *z* are the coordinates of the voxel, *I*(*x,y,z*) the indicator function indicating whether a voxel belongs to the parotid, and μ*_pqr_* central moments of the parotid. ȳ and $\bar{z}$ were defined analogously to $\bar{x}$.

### B Dose–Volume Histogram

#### B.1 Mean

The mean dose to the parotid gland.

#### B.2 Spread

The spread of the differential dose–volume histogram was quantified by the SD of the dose within the parotid gland.

#### B.3 Skewness

The skewness of the differential dose–volume histogram was measured by the third standardized moment. Negative skewness corresponds to the dose–volume histogram skewed toward lower dose, whereas positive skewness means the dose–volume histogram is skewed toward higher dose.

#### B.4 Dx

The minimum dose to *x*% “hottest” volume of the parotid gland.

#### B.5 Vx

Percentage volume of the parotid gland receiving at least *x* Gy.

#### B.6 Entropy

Entropy *H* measures smoothness of the dose within the parotid gland (45):

$$H=-\overset{256}{\underset{i=1}{\sum }}\;m\left(d_{i}\right)\;\log\;m\left(d_{i}\right),$$

where *d_i_* is the dose delivered to the *i*th voxel and *m*(*d_i_*) is the corresponding histogram. *H* = 0 for a uniform dose and *H* > 0 for a nonuniform dose.

#### B.7 Uniformity

Uniformity *U* of the dose within the parotid gland (45):

$$U=\overset{256}{\underset{i=1}{\sum }}\;m^{2}\left(d_{i}\right),$$

*U* = 1 for a uniform dose and *U* < 1 for a nonuniform dose.

### C Subvolume Mean Dose

Parotid gland subvolumes were defined by axial, coronal, and sagittal slices that cut parotid glands in thirds along the patient’s axes. The cuts were positioned in such a way that each subvolume comprised approximately the same number of voxels. As a result, nine, not exclusive, subvolumes were defined: three in x, three in y, and three in z direction. For each subvolume the mean radiation dose was calculated, e.g., the mean dose to the anterior third of the parotid gland ($s_{y}^{1}$) or the mean dose to the superior third of the parotid gland ($s_{z}^{3}$).

### D Dose Gradients

Average dose gradients measured average change of the dose along one of patient axes and were defined as:

$${\text{Gradient}}_{\text{x}}=\frac{\begin{array}{c} \sum_{x,y,z}\;D\left(x+1,y,z\right)I\left(x+1,y,z\right)\\ -D\left(x-1,y,z\right)I\left(x-1,y,z\right) \end{array}}{2\sum_{x,y,z}\;I\left(x,y,z\right)},$$

where *x*, *y*, and *z* are the coordinates of the voxel, *D*(*x,y,z*) the dose delivered to the voxel, and *I*(*x,y,z*) the indicator function indicating whether a voxel belongs to the parotid. Gradient_y_ and gradient_z_ were defined analogously to gradient_x_.

### E Three-Dimensional Dose Moments

The scale invariant dose moments allowed to quantify three-dimensional shape of the dose distribution within the parotid gland. Visualization of the moments can be found in Buettner et al. Supplementary Figure 1–3 (4). The moments were defined as:

$$\eta_{\mathrm{pqr}}=\frac{\sum_{x,y,z}\;{\left(x-\bar{x}\right)}^{p}{\left(y-ȳ\right)}^{q}{\left(z-\bar{z}\right)}^{r}D\left(x,y,z\right)I\left(x,y,z\right)}{{\left(\sum_{x,y,z}\;D\left(x,y,z\right)I\left(x,y,z\right)\right)}^{\frac{p+q+r}{3}+1}}$$

$$\bar{x}=\frac{\sum_{x,y,z}\;\mathrm{xI}\left(x,y,z\right)D\left(x,y,z\right)}{\sum_{x,y,z}\;I\left(x,y,z\right)D\left(x,y,z\right)},$$

ȳ and $\bar{z}$ were defined analogously. In particular, we considered moments quantifying dose variance, covariance, skewness, and coskewness.

#### E.1 Dose Variance (*η*_200_, *η*_020_, *η*_002_)

Dose variance corresponds to the spread of the dose along a given direction.

#### E.2 Dose Covariance (*η*_110_, *η*_101_, *η*_011_)

Dose covariance measures how the dose covaries along two axes. For example, positive values of *η*_110_ correspond to dose deposition along *xy* direction, whereas negative values correspond to dose deposition along the direction perpendicular to *xy*.

#### E.3 Dose Skewness (*η*_300_, *η*_030_, *η*_003_)

Dose skewness measures asymmetry of the dose distribution along a given axis.

#### E.4 Dose Coskewness (*η*_210_, *η*_201_, *η*_120_, *η*_021_, *η*_012_, *η*_102_)

Dose coskewness measures how dose variance along one direction covaries with another dimension, e.g., negative value of *η*_210_ would mean that variance of the dose along *x* axis increases when moving up the *y* axis.

## Appendix B

It has been reported that class imbalance together with low size of the minority class can hinder the performance of predictive models. There are two approaches commonly taken to alleviate this problem: oversampling and undersampling. In oversampling, one reduces the imbalance between classes by random replication or synthetic creation of minority class observations. Conversely, in undersampling the majority class size is reduced by elimination of its observations. Additionally, there are data cleaning methods which, through undersampling, aim to remove the observations that are considered noise or the observations close to the decision boundary, irrespective of their class membership. As a result, data cleaning methods do not reduce class imbalance but rather improve definitions of class clusters. Hyperparameters used to tune the sampling and the data cleaning algorithms are listed in Table A1.

**Table A1:** Hyperparameters used to tune the sampling algorithms.

Algorithm	Hyperparameters	Values
ROS	–	–
SMOTE	**k_neighbors**: Number of nearest neighbors used to construct synthetic samples.	{3,4,5}
	**m_neighbors**: Number of nearest neighbors used to determine if a minority sample is in danger.	{7,8,9}
	**kind**: Type of SMOTE algorithm.	{“regular,” “borderline1,” “borderline2”}
ADASYN	**n_neighbors**: Number of nearest neighbors to use to construct synthetic samples.	{3,5,8}
OSS	–	–
TL	–	–
ENN	**n_neighbors**: Number of nearest neighbors.	{2,3,5}
	**kind_sel**: Type of ENN algorithm.	{“all,” “mode”}
NCL	**n_neighbors**: Number of nearest neighbors.	{2,3,5}
SMOTE + TL	–	–
SMOTE + ENN	–	–

*Hyperparameters not listed in this table assumed the default values of imbalanced-learn package (38)*.

### A Random Oversampling

The data set imbalance is reduced by randomly duplicating observations from the minority class.

### B Synthetic Minority Oversampling

Synthetic minority oversampling (SMOTE) was proposed by Chawla et al. (46). The algorithm generates new synthetic minority observations by considering *k* nearest neighbors of a randomly selected minority observation. Next, the difference between the observation feature vector and one of the nearest neighbors feature vector is taken. This difference is then multiplied by a random weight between 0 and 1, and added to the observation feature vector to generate a new synthetic observation. In SMOTE, approximately equal number of synthetic observations is created for each minority class observation.

### C Adaptive Synthetic Sampling

Adaptive synthetic sampling (ADASYN) (47), similarly to SMOTE, generates synthetic minority class observations by interpolating feature vectors between a minority class observation and a randomly selected nearest neighbor. The key difference to SMOTE is that ADASYN aims to create more synthetic data for minority class observations that are hard to learn. For that reason, a learning difficulty weight is calculated for each minority class observation, based on the number of majority class observations in its neighborhood. Based on these weights, more synthetic observations are created for “difficult” minority class observations.

### D Tomek Links

A pair of observations (*E_i_,E_j_*) stemming from different classes and with distance *d*(*E_i_*, *E_j_*) form a Tomek link if there is no observation *E_l_*, such that *d*(*E_i_*, *E_l_*) < *d*(*E_i_*, *E_j_*) or *d*(*E_j_*, *E_l_*) < *d*(*E_i_*, *E_j_*) (48). As an undersampling method, all the observations in the majority class forming Tomek links are removed; when used as a data cleaning method, both the observation from the majority and the observation from the minority class are eliminated.

### E Condensed Nearest Neighbor Rule

The condensed nearest neighbor rule (CNN) proposed by Hart (49) undersamples the data set to find a consistent subset Ê of all observations *E*. First, all minority class observations and one randomly selected majority class observation are moved to Ê. Next, the rest of the majority class observations are classified using 1-nearest neighbor rule and during this process every misclassified observation is moved to subset Ê. The procedure continues until all misclassified observations are in the subset Ê (50). Intuitively, CNN reduces the number of redundant observations in majority class that are far from the decision border and therefore less informative in learning.

### F One-Sided Selection

One-sided selection (OSS) (50) is an undersampling method realized by Tomek links algorithm followed by CNN. Tomek links undersample the majority class and remove noisy and borderline class observations. CNN, on the other hand, removes observations from the majority class that are distant from the decision border and likely are not informative.

### G Wilson’s Edited Nearest Neighbor Rule

The Wilson’s edited nearest neighbor rule (ENN) (51) removes all observations which class label differ from the class of its *k* nearest neighbors.

### H Neighborhood Cleaning Rule

The neighborhood cleaning rule (NCL) (52) is a modification of the ENN algorithm. As in the ENN, the class of each observation is compared with the classes of its *k* nearest neighbors. If the analyzed observation belongs to the majority class, the procedure is the same as in the ENN. However, if the observation belongs to the minority class and its *k* nearest neighbors to the majority class, the minority class observation is kept in the data set and the *k* nearest neighbors are removed.

### I SMOTE + TL

First, the original data set is oversampled with SMOTE, and then Tomek links are identified and removed. The method aims to produce a balanced data set with well-defined class clusters (53).

### J SMOTE + ENN

This method is similar to SMOTE + TL but with stronger data cleaning component realized by the ENN (53).

## Appendix C

Feature selection is a crucial part of model building. It not only allows to improve accuracy of model predictions but also reduces the dimensionality of the input space. A reduced dimensionality of the input space decreases the risk of model overfitting and improves model interpretability. Hyperparameters used to tune the feature selection algorithms are listed in Table A2.

**Table A2:** Hyperparameters used to tune the feature selection algorithms.

Algorithm	Hyperparameters	Values
UFS-F	**k**: Number of features to select.	{2,3,4,5,6}
UFS-MI	**k**: Number of features to select.	{2,3,4,5,6}
RFE-LR	**k**: Number of features to select.	{2,3,4,5,6}
	**step**: Number of features to	1
	remove at each iteration.	
	**class_weight**: Whether class weights	{None, “balanced”}
	are equal or inversely proportional
	to class frequencies.
	**C**: Inverse of regularization strength.	{2^−5^, 2^−4.985^,
		2^−4.97^, …, 2^10^}
	**penalty**: Type of regularization.	“l2”
RFE-ET	**k**: Number of features to select.	{2,3,4,5,6}
	**step**: Fraction of features to remove	0.5
	at each iteration.	
	**class_weight**: Whether class weights	{None, “balanced,”
	are equal or inversely proportional to	“balanced_subsample”}
	class frequencies.	
	**n_estimators**: Number of	[90,140]
	decision trees.	
MB-LR	**k**: Number of features to select.	{2,3,4,5,6}
	**class_weight**: Whether class weights	{None, “balanced”}
	are equal or inversely proportional to
	class frequencies.
	**C**: Inverse of regularization strength.	{2^−5^, 2^−4.985^,
		2^−4.97^, …, 2^10^}
	**penalty**: Type of regularization.	{“l1,” “l2”}
MB-ET	**k**: Number of features to select.	{2,3,4,5,6}
	**class_weight**: Whether class weights	{None, “balanced,”
	are equal or inversely proportional	“balanced_subsample”}
	to class frequencies.	
	**n_estimators**: Number of	[90,140]
	decision trees.	

*Hyperparameters not listed in this table assumed the default values of scikit-learn package (43)*.

**Table A3:** Hyperparameters used to tune the classification algorithms.

Algorithm	Hyperparameters	Values
LR-L1	**class_weight**: Whether class weights are equal or inversely proportional to class frequencies.	{None, “balanced”}
	**C**: Inverse of regularization strength.	{2^−5^, 2^−4.985^, 2^−4.97^, …, 2^10^}
LR-L2	**class_weight**: Whether class weights are equal or inversely proportional to class frequencies.	{None, “balanced”}
	**C**: Inverse of regularization strength.	{2^−5^, 2^−4.985^, 2^−4.97^, …, 2^10^}
LR-EN	**class_weight**: Whether class weights are equal or inversely proportional to class frequencies.	{None, “balanced”}
	**alpha**: Regularization strength.	{2^−10^, 2^−9.985^, 2^−9.97^, …, 2^5^}
	**l1_ratio**: Ratio between L1 and L2 penalty.	[0,1]
kNN	**n_neighbors**: Number of nearest neighbors.	{1,2,3,…,9}
	**p**: Power parameter of the Minkowski distance.	{1,2,∞}
SVM	**class_weight**: Whether class weights are equal or inversely proportional to class frequencies.	{None, “balanced”}
	**C**: Inverse of regularization strength.	{2^−5^, 2^−4.985^, 2^−4.97^, …, 2^10^}
	**gamma**: Parameter of the RBF kernel.	{2^−15^, 2^−14.982^, 2^−14.964^, …, 2^3^}
ET	**n_estimators**: Number of decision trees.	[90, 230]
	**class_weight**: Whether class weights are equal of inversely proportional to class frequencies.	{None, “balanced”}
	**criterion**: The function to measure the quality of a split.	{“gini,” “entropy”}
	**max_features**: Number of features to consider when calculating the best split.	{0.05, 0.10, 0.15,…,1}
	**min_samples_split**: The minimum number of samples required to split a node.	{2,3,4,…,20}
	**min_samples_leaf**: The minimum number of samples required to be at a leaf node.	{1,2,3,…,20}
GTB	**n_estimators**: Number of decision trees.	[200, 2000]
	**learning_rate**: Boosting learning rate.	{2^−7^, 2^−6.994^, 2^−6.988^, …, 2^−1^}
	**max_depth**: Maximum tree depth.	{1,2,3,…,6}
	**gamma**: Minimum loss reduction required to make a further partition on a leaf node of the tree.	{0.05,0.1,0.3,0.5,0.7,0.9,1}
	**min_child_weight**: Minimum sum of instance weight(hessian) needed in a child.	{1,3,5,7}
	**subsample**: Ratio of the training samples used to grow trees.	{0.6,0.65,0.70,…,1}
	**reg_lambda**: L1 regularization term on weights.	[0,1]
	**reg_alpha**: L2 regularization term on weights.	[0,1]

*Hyperparameters not listed in this table assumed the default values of scikit-learn (43) and xgboost (44) packages*.

### A Univariate Feature Selection

Univariate feature selection methods evaluate each feature separately relying solely on the relation between one feature characteristic and the modeled variable. After all the features were graded, the features with the highest rankings are selected. A disadvantage of univariate feature selection is that the algorithm fails to select features which have relatively low individual scores but a high score when combined together. Also, due to the fact that univariate feature selection methods evaluate features individually, they are unable to handle feature redundancy (54, 55).

#### A.1 Fisher Score

Intuitively, Fisher score is a ratio of the between-class scatter to the within-class scatter. As a result, high Fisher scores correspond to features with well defined class clusters (low within-class scatter) that are distant from each other (large between-class scatter) (56). Fisher score is commonly used in supervised classification tasks due to its low computational cost and general good performance (54).

Fisher score of feature *X* was calculated using the following formula (57):

$$F\left(X\right)=\frac{\frac{1}{C-1}\sum_{c=1}^{C}\;N_{c}{\left({\bar{x}}_{c}-\bar{x}\right)}^{2}}{1N-C\sum_{c=1}^{C}\;\sum_{i:y_{i}=c}\;{\left(x_{i}-{\bar{x}}_{c}\right)}^{2}}$$

$$\bar{x}=\frac{1}{N}\overset{N}{\underset{i=1}{\sum }}\;x_{i}$$

$${\bar{x}}_{c}=\frac{1}{N_{c}}\underset{i:y_{i}=c}{\sum }\;x_{i},$$

where *C* is the number of classes, *N* total number of observations, *N_c_* number of observations in class *c*, $\bar{x}$ mean value of feature *X*, and ${\bar{x}}_{c}$ mean value of feature *X* in class *c*.

#### A.2 Mutual Information

This univariate feature selection method measures mutual information between each feature and the modeled variable. Intuitively, mutual information measures how much knowing the feature *X* value reduces uncertainty about the class label *Y*, and vice versa (58). This can be expressed by the formula:

MI(X;Y)=H(X)−H(X|Y)=H(Y)−H(Y|X),

where *H(X)* is the entropy of *X* and *H(X|Y)* is the entropy of *X* after observing class *Y*.

$$H\left(X\right)=-\overset{N}{\underset{i=1}{\sum }}\;p\left(x_{i}\right)\log p\left(x_{i}\right)$$

$$H\left(X|Y\right)=-\overset{N}{\underset{i=1}{\sum }}\;p\left(y_{i}\right)\overset{N}{\underset{k=1}{\sum }}\;p\left(x_{k}|y_{i}\right)\log p\left(x_{k}|y_{i}\right).$$

Features with high mutual information are considered informative and are selected.

### B Recursive Feature Elimination

In the first step of recursive feature elimination (RFE), an induction algorithm is trained using the full set of features. Next, the features are ranked according to a given criterion, such as feature weight in logistic regression or feature importance in ensemble models. Then, the feature or the features with the smallest ranks are removed from the feature set. This procedure is repeated iteratively until the desired number of features is achieved (59, 60).

In contrast to univariate feature selection, recursive feature elimination methods can capture feature interactions. For that reason it can select not only good univariate predictors but also features which have low predictive power alone but high predictive power when pooled together.

The ability to handle feature redundancy depends on the induction algorithm used with RFE. For instance, L1-penalized logistic regression tends to select one of highly correlated features, hence reducing feature redundancy (61). On the contrary, L2-penalized logistic regression tends to give similar weights to correlated features, distributing the total feature importance among them. For the recursive feature elimination, we used two induction algorithms: logistic regression and extra-trees.

### C Model-Based Feature Selection

Model-based feature selection can be considered a special case of recursive feature elimination with only one iteration step. The induction algorithm is trained using the full set of features and the desired number of lowest scoring features is removed. Similarly to RFE, we employed logistic regression and extra-trees as the induction algorithms.

## Appendix D

The selection of the classifier is a critical part of model building, which directly determines the flexibility of the decision boundary. On the one hand, a too flexible model can result in overfitting and low generalizability. On the other hand, a too simple model can fail to capture the complexity of the true decision boundary and result in underfitting. Furthermore, the interpretability of the model depends strongly on the type of the chosen algorithm. Hyperparameters used to tune the classification algorithms are listed in Table A3.

### A Logistic Regression

Logistic regression is a simple linear model allowing to estimate probability of a binary response based on a number of risk factors. In order to avoid overfitting, logistic regression is usually regularized *via* L1, L2, or elastic net penalty. L1 penalty outperforms L2 penalty in terms of handling irrelevant and redundant features (62). Its ability to bring feature weights to zero results in sparse models and improves model interpretability (63). On the other hand, L1 tends to randomly select one of highly correlated features which can result in model variability (64). The elastic net method brings in a way the two worlds together and applies a penalty that is a convex combination of L1 and L2 regularization (64).

The advantages of logistic regression are its simplicity, interpretability, and easy tuning (only one hyperparameter with L1 or L2 regularization or two hyperparameters with elastic net regularization). The biggest disadvantage is a linear hypersurface decision boundary that may not be flexible enough to describe the real decision boundary.

### B k-Nearest Neighbors

The k-nearest neighbor (kNN) classifier looks at the *k* points in the training set that are nearest to the test input. The object is classified based on a majority vote of its neighbors (58). kNN has a much more flexible decision boundary compared to logistic regression. It will likely outperform logistic regression when the true decision boundary is highly irregular. Nevertheless, the curse of dimensionality has a considerable impact on the performance of the k-nearest neighbors classifier making feature selection crucial when working with high-dimensional data sets.

### C Support Vector Machine

Similarly to the k-nearest neighbors algorithm, the support vector machine does not learn a fixed set of parameters corresponding to the features of the input. It rather remembers the training examples and classifies new observations based on some similarity function. The two main concepts behind support vector machines are the kernel trick and the large margin principle. The kernel trick guarantees high flexibility of the decision boundary by allowing to operate in feature spaces of very high, even infinite, dimensionality. The large margin principle ensures model sparsity by discarding all observations not laying on maximum margin hypersurfaces. Support vector machines proved to be very successful in various classification tasks, including NTCP modeling. Unfortunately, interpretation of support vector machines with nonlinear kernels is a challenge (65).

### D Extra-Trees

The extra-trees classifier is an ensemble of decision trees. Each tree is built either on the full learning sample or on a bootstrap replica. At each node, a random subset of features is selected and for each feature a random cut-point is drawn. The best feature-cutpoint pair is selected to split the node. The tree is grown until the minimum sample size for splitting a node is reached. The ensemble predictions are the results of the majority vote of predictions of individual trees (66). A big advantage of the extra-trees algorithm is that it works “out-of-the-box” with no or minimal hyperparameter tuning.

### E Gradient Tree Boosting

Similarly to extra-trees, gradient tree boosting uses an ensemble of decision trees. Gradient tree boosting iteratively fits small decision trees to the data set in an adaptive fashion. After each iteration, training samples are reweighted to focus on the instances misclassified by the previous trees. When all trees are grown, the prediction is obtained by the weighted majority vote of the trees (61, 67).

Gradient tree boosting proved to be a very successful algorithm often outperforming neural networks, support vector machines, and other ensemble models. However, tuning the hyperparameters may be challenging.

## References

[1] Deasy JO Moiseenko V Marks L Chao KSC Nam J Eisbruch A. Radiotherapy dose-volume effects on salivary gland function. Int J Radiat Oncol Biol Phys (2010) 76(3 Suppl):58–63. 10.1016/j.ijrobp.2009.06.090 20171519 PMC4041494

[2] Houweling AC Philippens MEP Dijkema T Roesink JM Terhaard CHJ Schilstra C A comparison of dose-response models for the parotid gland in a large group of head-and-neck cancer patients. Int J Radiat Oncol Biol Phys (2010) 76(4):1259–65. 10.1016/j.ijrobp.2009.07.1685 20005639

[3] Beetz I Schilstra C Burlage FR Koken PW Doornaert P Bijl HP Development of NTCP models for head and neck cancer patients treated with three-dimensional conformal radiotherapy for xerostomia and sticky saliva: the role of dosimetric and clinical factors. Radiother Oncol (2012) 105(1):86–93. 10.1016/j.radonc.2011.05.010 21632133

[4] Buettner F Miah AB Gulliford SL Hall E Harrington KJ Webb S Novel approaches to improve the therapeutic index of head and neck radiotherapy: an analysis of data from the PARSPORT randomised phase III trial. Radiother Oncol (2012) 103(1):82–7. 10.1016/j.radonc.2012.02.006 22444242

[5] Lee T-F Liou M-H Ting H-M Chang L Lee H-Y Wan Leung S Patient- and therapy-related factors associated with the incidence of xerostomia in nasopharyngeal carcinoma patients receiving parotid-sparing helical tomotherapy. Sci Rep (2015) 5:13165. 10.1038/srep13165 26289304 PMC4542473

[6] Gabrys HS Buettner F Sterzing F Hauswald H Bangert M. Parotid gland mean dose as a xerostomia predictor in low-dose domains. Acta Oncol (2017) 56(9):1197–203. 10.1080/0284186X.2017.1324209 28502238

[7] Eisbruch A Kim HM Terrell JE Marsh LH Dawson LA Ship JA. Xerostomia and its predictors following parotid-sparing irradiation of head-and-neck cancer. Int J Radiat Oncol Biol Phys (2001) 50(3):695–704. 10.1016/S0360-3016(01)01512-7 11395238

[8] Lee T-F Chao PJ Ting HM Chang L Huang YJ Wu JM Using multivariate regression model with least absolute shrinkage and selection operator (LASSO) to predict the incidence of xerostomia after intensity-modulated radiotherapy for head and neck cancer. PLoS One (2014) 9(2):e89700 10.1371/journal.pone.0089700 24586971 PMC3938504

[9] Hawkins PG Lee JY Mao Y Li P Green M Worden FP Sparing all salivary glands with IMRT for head and neck cancer: longitudinal study of patient-reported xerostomia and head-and-neck quality of life. Radiother Oncol (2018) 126(1):68–74. 10.1016/j.radonc.2017.08.002 28823405

[10] Luijk PV Pringle S Deasy JO Moiseenko VV Faber H Hovan A Sparing the region of the salivary gland containing stem cells preserves saliva production after radiotherapy for head and neck cancer. Sci Transl Med (2015) 7(305):1–8. 10.1126/scitranslmed.aac4441 26378247 PMC4964284

[11] van Dijk LV Brouwer CL van der Schaaf A Burgerhof JGM Beukinga RJ Langendijk JA CT image biomarkers to improve patient-specific prediction of radiation-induced xerostomia and sticky saliva. Radiother Oncol (2017) 122(2):185–91. 10.1016/j.radonc.2016.07.007 27459902

[12] van Dijk LV Brouwer CL Paul H Laan VD Johannes GM Langendijk JA Geometric image biomarker changes of the parotid gland are associated with late xerostomia. Int J Radiat Oncol Biol Phys (2017) 99(5):1101–10. 10.1016/j.ijrobp.2017.08.003 28939226

[13] El Naqa I Bradley JD Lindsay PE Hope AJ Deasy JO. Predicting radiotherapy outcomes using statistical learning techniques. Phys Med Biol (2009) 54(18):S9–30. 10.1088/0031-9155/54/18/S02 19687564 PMC4041524

[14] Gulliford S Modelling of normal tissue complication probabilities (NTCP): review of application of machine learning in predicting NTCP. In: El Naqa I Li R Murphy MJ, editors. Machine Learning in Radiation Oncology. Cham: Springer (2015). p. 277–310.

[15] Dean JA Welsh LC Wong KH Aleksic A Dunne E Islam MR Normal tissue complication probability (NTCP) modelling of severe acute mucositis using a novel oral mucosal surface organ at risk. Clin Oncol (2017) 29(4):263–73. 10.1016/j.clon.2016.12.001 28057404 PMC6175048

[16] Chen S Zhou S Yin F-F Marks LB Das SK. Investigation of the support vector machine algorithm to predict lung radiation-induced pneumonitis. Med Phys (2007) 34(10):3808–14. 10.1118/1.2776669 17985626 PMC2920285

[17] Ospina JD Zhu J Chira C Bossi A Delobel JB Beckendorf V Random forests to predict rectal toxicity following prostate cancer radiation therapy. Int J Radiat Oncol Biol Phys (2014) 89(5):1024–31. 10.1016/j.ijrobp.2014.04.027 25035205

[18] Statnikov A Aliferis CF Tsamardinos I Hardin D Levy S. A comprehensive evaluation of multicategory classification methods for microarray gene expression cancer diagnosis. Bioinformatics (2005) 21(5):631–43. 10.1093/bioinformatics/bti033 15374862

[19] Olson RS La Cava W Mustahsan Z Varik A Moore JH Data-Driven Advice for Applying Machine Learning to Bioinformatics Problems. (2017). *ArXiv*. PMC5890912 29218881

[20] Parmar C Grossmann P Rietveld D Rietbergen MM Lambin P Aerts HJWL Radiomic machine learning classifiers for prognostic biomarkers of head & neck cancer. Front Oncol (2015) 5:272 10.3389/fonc.2015.00272 26697407 PMC4668290

[21] National Cancer Institute (U.S.). Common Terminology Criteria for Adverse Events (CTCAE) v4.03. Bethesda, MD: U.S. Department of Health and Human Services (2010).

[22] Salkind NJ Encyclopedia of Measurement and Statistics. Thousand Oaks: SAGE Publications (2007). p. 508–10.

[23] Eisbruch A Ten Haken RK Kim HM Marsh LH Ship JA. Dose, volume, and function relationships in parotid salivary glands following conformal and intensity-modulated irradiation of head and neck cancer. Int J Radiat Oncol Biol Phys (1999) 45(3):577–87. 10.1016/S0360-3016(99)90269-9 10524409

[24] Roesink JM Moerland MA Battermann JJ Hordijk GJ Terhaard CH. Quantitative dose-volume response analysis of changes in parotid gland function after radiotherapy in the head-and-neck region. Int J Radiat Oncol Biol Phys (2001) 51(4):938–46. 10.1016/S0360-3016(01)01717-5 11704314

[25] Hanley JA McNeil BJ The meaning and use of the area under a receiver characteristic (ROC) curve. Radiology (1982) 143:29–36. 10.1148/radiology.143.1.7063747 7063747

[26] Qin G Hotilovac L. Comparison of non-parametric confidence intervals for the area under the ROC curve of a continuous-scale diagnostic test. Stat Methods Med Res (2008) 17(2):207–21. 10.1177/0962280207087173 18426855

[27] Benjamini Y Hochberg Y Controlling the false discovery rate: a practical and powerful approach to multiple testing. J R Stat Soc B (1995) 57(1):289–300.

[28] Gavrilov Y Benjamini Y Sarkar SK An adaptive step-down procedure with proven FDR control under independence. Ann Stat (2009) 37(2):619–29. 10.1214/07-AOS586

[29] Japkowicz N Stephen S The class imbalance problem: a systematic study. Intell Data Anal (2002) 6(5):429–49.

[30] He H Garcia EA Learning from imbalanced data. IEEE Trans Knowl Data Eng (2009) 21(9):1263–84. 10.1109/TKDE.2008.239

[31] Guyon I Elisseeff A An introduction to variable and feature selection. J Mach Learn Res (2003) 3:1157–82.

[32] Bergstra J Bengio Y Random search for hyper-parameter optimization. J Mach Learn Res (2012) 13:281–305.

[33] Molinaro AM Simon R Pfeiffer RM. Prediction error estimation: a comparison of resampling methods. Bioinformatics (2005) 21(15):3301–7. 10.1093/bioinformatics/bti499 15905277

[34] Krzanowski W Hand D Assessing error rate estimators: the leave-one-out method reconsidered. Aust N Z J Stat (1997) 39(1):35–46. 10.1111/j.1467-842X.1997.tb00521.x

[35] Airola A Pahikkala T Waegeman W De Baets B Salakoski T An experimental comparison of cross-validation techniques for estimating the area under the ROC curve. Comput Stat Data Anal (2011) 55(4):1828–44. 10.1016/j.csda.2010.11.018

[36] Holm S A simple sequentially rejective multiple test procedure. Scand J Stat (1979) 6:65–70.

[37] Cawley GC Talbot NLC On over-fitting in model selection and subsequent selection bias in performance evaluation. J Mach Learn Res (2010) 11:2079–107.

[38] Lemaitre G Nogueira F Aridas CK Imbalanced-learn: a Python toolbox to tackle the curse of imbalanced datasets in machine learning. J Mach Learn Res (2017) 18(17):1–5.

[39] Hunter JD Matplotlib: a 2D graphics environment. Comput Sci Eng (2007) 9(3):99–104. 10.1109/MCSE.2007.55

[40] Van Der Walt S Colbert SC Varoquaux G The NumPy array: a structure for efficient numerical computation. Comput Sci Eng (2011) 13(2):22–30. 10.1109/MCSE.2011.37

[41] Demšar J Curk T Erjavec A Hočevar T Milutinovič M Možina M Orange: data mining toolbox in Python. J Mach Learn Res (2013) 14:2349–53.

[42] McKinney W Data structures for statistical computing in Python. In: van der Walt S Millman J, editors. SciPy 2010: Proceedings of the 9th Python in Science Conference Austin, TX, USA (2001) p. 51–6.

[43] Pedregosa F Varoquaux G Gramfort A Michel V Thirion B Grisel O Scikit-learn: machine learning in Python. J Mach Learn Res (2011) 12:2825–30.

[44] Chen T Guestrin C XGBoost: A Scalable Tree Boosting System. (2016). p. 1–6. arXiv Prepr. arXiv1603.02754v3.

[45] Gonzalez RC Woods RE Digital Image Processing. 3rd ed Upper Saddle River, NJ: Prentice-Hall, Inc (2006).

[46] Chawla NV Bowyer KW Hall LO Kegelmeyer WP SMOTE: synthetic minority over-sampling technique. J Artif Intell Res (2002) 16:321–57.

[47] He H Bai Y Garcia EA Li S ADASYN: adaptive synthetic sampling approach for imbalanced learning. In Proc 2008 IEEE International Joint Conference on Neural Networks (IEEE World Congress on Computational Intelligence) Hong Kong, China (2008). p. 1322–8.

[48] Tomek I Two modifications of CNN. IEEE Trans Syst Man Cybern (1976) 6:769–72.

[49] Hart PE The condensed nearest neighbour rule. IEEE Trans Inf Theory (1968) 14(5):515–6. 10.1109/TIT.1968.1054155

[50] Kubat M Matwin S Addressing the course of imbalanced training sets: one-sided selection. In: Fisher DH, editor. Proceedings of the Fourteenth International Conference on Machine Learning (ICML) Nashville, TN, USA/San Francisco: Morgan Kaufmann (1997). p. 179–86.

[51] Wilson DR Asymptotic properties of nearest neighbor rules using edited data. Inst Electr Electron Eng Trans Syst Man Cybern (1972) 2(3):408–21.

[52] Laurikkala J Improving identification of difficult small classes by balancing class distribution. In: Quaglini S Barahona P Andreassen S, editors. AIME 2001 Artificial Intelligence in Medicine: Proceedings of the 8th Conference on Artificial Intelligence in Medicine in Europe Cascais, Portugal/Berlin: Springer (2001) p. 63–6.

[53] Batista GEAPA Prati RC Monard MC A study of the behavior of several methods for balancing machine learning training data. ACM Sigkdd Explor Newsl (2004) 6(1):20–9. 10.1145/1007730.1007735

[54] Gu Q Li Z Han J Generalized Fisher Score for feature selection. CoRR (2012) 3:327–30.

[55] Tang J Alelyani S Liu H Feature selection for classification: a review. In: Aggarwal CC, editor. Data Classification Algorithms and Applications. Boca Raton, FL: CRC Press (2014). p. 37–64.

[56] Duda RO Hart PE Stork DG Pattern Classification. New York, NY: John Wiley and Sons (2012).

[57] Lowry R, editor. One-way analysis of variance for independent samples. Concepts and Applications of Inferential Statistics. Poughkeepsie, NY: DOER – Directory of Open Educational Resources (2014).

[58] Murphy KP Machine Learning: A Probabilistic Perspective. Cambridge, MA: The MIT Press (2012).

[59] Kohavi R John G Wrappers for feature subset selection. Artif Intell (1997) 97(97):273–324. 10.1016/S0004-3702(97)00043-X

[60] Guyon I Weston J Barnhill S Vapnik V Gene selection for cancer classification using support vector machines. Mach Learn (2002) 46(1–3):389–422. 10.1023/A:1012487302797

[61] Hastie T Tibshirani RJ Friedman J The Elements of Statistical Learning: Data Mining, Inference and Prediction. 2 ed New York, NY: Springer (2009).

[62] Ng AY Feature selection, L1 vs. L2 regularization, and rotational invariance. In: Brodley C, editor. ICML 2004: Proceedings of the Twenty-First International Conference on Machine Learning Banff, Alberta, Canada/New York: ACM (2004). 78 p.

[63] Bishop CM Pattern Recognition and Machine Learning. 1 ed New York, NY: Springer (2006).

[64] Zou H Hastie T Regularization and variable selection via the elastic net. J R Stat Soc B (2005) 67:301–20. 10.1111/j.1467-9868.2005.00527.x

[65] Burges CJC A tutorial on support vector machines for pattern recognition. Data Min Knowl Discov (1998) 2:121–67. 10.1023/A:1009715923555

[66] Geurts P Ernst D Wehenkel L Extremely randomized trees. Mach Learn (2006) 63(1):3–42. 10.1007/s10994-006-6226-1

[67] Freund Y Schapire RE A decision-theoretic generalization of on-line learning and an application to boosting. J Comput Syst Sci (1997) 55(1):119–39. 10.1006/jcss.1997.1504

